# Investigating the Alkaline Potential of Mineral Trioxide Aggregate Repair Using Selenium Nanoparticles

**DOI:** 10.1590/0103-6440202405760

**Published:** 2024-06-24

**Authors:** Njwan Fadhel Shehab, Nadia Hameed Hasan, Hana Khaleel Ismail

**Affiliations:** 1 Department of Conservative Dentistry/ College of Dentistry/University of Mosul Iraq; 2 Department of pathology and poultry disease/College of Veterinary medicine/University of Mosul Iraq

**Keywords:** Selenium Nanoparticles, Mineral Trioxide Aggregate Repair High Plasticity, Alkalinizing Activity, FE-SEM and EDX Analysis

## Abstract

This study aimed to determine the effect of adding selenium nanoparticles (SeNPs) to mineral trioxide aggregate (MTA HP) concerning alkalinizing potential. Additionally, it examined the set material after SeNPs incorporation using Field Emission Scanning Electron Microscopy with Energy Dispersive X-ray analysis (FE-SEM/EDX) for characterizing the elemental composition and morphological alterations resulting from the integration of SeNPs. Cement samples, both before and after SeNPs incorporation, were examined using FE-SEM/EDX. The pH level was also measured with a pH-meter previously calibrated with solutions of known pH, to evaluate the alkalinizing activity of the integrated substance at different concentrations of nanoparticles: Group 1 (control): 0% w/w SeNPs, Group 2: 0.5% w/w SeNPs, Group 3: 1% w/w SeNPs, Group 4: 1.5% w/w SeNPs and Group 5: 2% w/w SeNPs after 1, 7, 14, and 30 days in distal water. The data were analyzed by one-way ANOVA and Tukey tests (P≤0.05). According to FE-SEM/EDX, the morphological characteristics indicate that SeNPs were successfully dispersed and integrated into the MTA repair matrix. EDX examination validates the presence of Selenium, confirming successful integration. The findings confirmed that the MTAHP showed a high pH level with a discernible reduction in the alkalinizing activity with each incorporated concentration of (SeNPs) that significantly differed from the control group across various periods at (P≤ 0.05). Consequently, the findings indicate that the addition of SeNPs to MTA HP has a notable impact on the pH of the storage solution, leading to a significant decrease in pH values for all concentrations and periods when compared to the control group. The alkalinizing action of MTA HP is highly affected by the incorporated SeNPs, making it more suitable for application in pulpal tissue. This study contributes to our understanding of the morphological alterations and elemental composition of SeNP-incorporated MTA HP, enhancing its potential applications in dental and tissue regeneration.

## Resumo

Este estudo teve como objetivo determinar o efeito da adição de nanopartículas de selênio (SeNPs) ao agregado de trióxido mineral (MTA HP) em relação ao potencial alcalinizante. Além disso, examinou o material do conjunto após a incorporação de SeNPs usando a microscopia eletrônica de varredura de emissão de campo com análise de raios X por dispersão de energia (FE-SEM/EDX) para caracterizar a composição elementar e as alterações morfológicas resultantes da integração de SeNPs. As amostras de cimento, antes e depois da incorporação de SeNPs, foram examinadas usando FE-SEM/EDX. O nível de pH também foi medido com um pH-metro previamente calibrado com soluções de pH conhecido, para avaliar a atividade alcalinizante da substância integrada em diferentes concentrações de nanopartículas: Grupo 1 (controle): 0% p/p de SeNPs, Grupo 2: 0,5% p/p de SeNPs, Grupo 3: 1% p/p de SeNPs, Grupo 4: 1,5% p/p de SeNPs e Grupo 5: 2% p/p de SeNPs após 1, 7, 14 e 30 dias em água distal. Os dados foram analisados por ANOVA unidirecional e testes de Tukey (P≤0,05). De acordo com o FE-SEM/EDX, as características morfológicas indicam que as SeNPs foram dispersas e integradas com sucesso na matriz de reparo do MTA. A análise de EDX valida a presença de selênio, confirmando a integração bem-sucedida. Os resultados confirmaram que o MTAHP apresentou alto nível de pH com uma redução perceptível na atividade alcalinizante com cada concentração incorporada de (SeNPs) que diferiu significativamente do grupo de controle em vários períodos de tempo (P≤ 0,05). Consequentemente, os resultados indicam que a adição de SeNPs ao MTA HP tem um impacto notável sobre o pH da solução de armazenamento, levando a uma redução significativa nos valores de pH para todas as concentrações e períodos de tempo quando comparados ao grupo de controle. A ação alcalinizante do MTA HP é altamente afetada pelas SeNPs incorporadas, tornando-o mais adequado para aplicação no tecido pulpar. Este estudo contribui para a nossa compreensão das alterações morfológicas e da composição elementar do MTA HP incorporado com SeNPs, aprimorando suas possíveis aplicações na regeneração dentária e tecidual.

## Introduction

MTA cements, which are widely utilized in a variety of dental applications, are largely constructed of calcium silicate-based materials with the addition of a radiopacifier ^1^. Studies have demonstrated that relatively small changes in chemical composition, additions, or the radiopacifying agent ^2^ can dramatically affect the physicochemical behavior of these materials. Recent developments in MTA formulations have resulted in the creation of new versions, such as MTA Repair HP (High Plasticity) by Angelus, Brazil. MTA Repair HP is a bioceramic material with high plasticity that contains tricalcium silicate, dicalcium silicate, tricalcium aluminate, calcium oxide, and calcium tungstate. MTA Repair HP is a newly developed material produced by the same company as MTA Angelus. Its powder shares a chemical composition resembling that of MTA Angelus, but the radiopacifying ingredient is calcium tungstate instead of bismuth oxide in this novel formulation^3^. Notably, MTA Repair HP includes organic plasticizer polyvinylpyrrolidone (PVP) in the distilled water used for material hydration, serving as a plasticizer for enhancing its handling properties ^4^. These changes were made to enhance the material's performance and reduce problems such as tooth discoloration brought on by the reaction of bismuth oxide with dentin collagen ^3^^,^^5^.

MTA has a variety of uses in dental operations, including apexification, root and furcal perforation repair, and direct pulp capping ^6^. For MTA to set, sterile water must be mixed with the powder, which, through the action of water sorption, creates a colloidal gel, powder-to-liquid ratio, ambient moisture, MTA type, pH, and temperature, all have an impact on the characteristics of this gel ^7^. One of the most important properties of MTA is its capacity to release calcium ions and maintain a high pH alkalinizing activity, which is necessary for promoting the creation of hard tissue and demonstrating antibacterial activity. Furthermore, when in contact with phosphate-containing fluids, the continual release of calcium ions and high pH promotes the spontaneous development of a calcium phosphate apatite layer ^2^. However, the initial inflammatory response brought on by trauma and the toxic effects of substances is partially related to the high alkalinity created by calcium-silicate-based cement, which causes damage to nearby tissues (7, 8). Additionally, the presence of necrotic tissue suggests that MTA, similarly to calcium hydroxide, initially causes coagulation necrosis when in contact with the pulp tissue due to its high alkalinity^9^. Given the importance of calcium ions in the development of hard tissues in dental materials, as well as the need to minimize the harm caused by prolonged exposure to high pH, it is important to note that biomaterials used for dentin pulp capping stimulate the release of calcium ions. As a result, this stimulation helps the injured area produce calcium carbonate, which accelerates the process of mineralization ^10^.

Due to the unique properties of SeNPs they have gained attention in various fields, including dentistry. SeNPs exhibit excellent biocompatibility and antioxidant activity, which can contribute to tissue healing and regeneration. Moreover, SeNPs have demonstrated antimicrobial effects against common oral pathogens, making them suitable candidates for dental applications ^11^. The effect on pH value of the addition of SeNPs has previously not been tested. Therefore, the purpose of this study is to assess the pH and elemental analysis of calcium ions in experimental formulations of MTA Repair HP incorporated with green-synthesized SeNPs. A comparison with commercially available MTA Repair HP without SeNPs was performed. This work aims to give useful insights into increasing biocompatibility and overall performance in dental restorative materials by examining the alkalinizing activity of these novel MTA formulations, potentially improving patient outcomes. This study hypothesizes that SeNPs have an impact on the alkalinizing activity of MTA HP.

## Materials and Methods

### Nano-Selenium Particle-Modified Mineral Trioxide Aggregate (MTA) Preparation

With the use of a precision four-digit balance, nano-sized selenium particles were precisely measured and then added to MTA powder. Following the complete mixing of the powder of MTA with the powder of SeNPs, the resulting mixture was encapsulated and shaken for 5 seconds with a dental amalgamator or shaker. For the hydrated material preparation, in accordance with the manufacturer's instructions, the substance-specific liquid was added to the powder on a glass slab, mixed manually using a metallic spatula for blending the cement to obtain homogeneous consistency that was subsequently kept in an incubator at 37°C with a relative humidity of 95%. The set material was crushed into a fine powder using a mortar and pestle to test material qualities pre- and post-integration of SeNPs.

### Field Emission Scanning Electron Microscopy (FESEM) with Energy Dispersive X-Ray (EDX) Analysis

To evaluate the morphology, shape, and size of the nanoparticles as well as elemental analyses, the material underwent thorough characterization using FESEM/EDX (TE SCAN MIRA3, France). The FESEM was precisely calibrated to run at 15.0 kV accelerating voltage and 10 mA for image capture. The sample was covered in an aluminum coating that was 15 nm thin to improve imaging precision. Additionally, the experiment included observing and analyzing the hydrated material before and after the incorporation of SeNPs. These tests were carried out with the use of computer-controlled software. 40 particles were counted to determine the particle size distribution.

### XRD Analysis

XRD analysis was performed on the samples using a Phillips Xpert PANanalytical X-ray diffractometer from Holland. A Cu K radiation source (λ = 1.54060 A˚) was utilized and the study included a diffraction angle range (2θ) of 10 to 80 with a step size of 0.0500°. Each scan step in the diffractometer took 1.0000 seconds, and it ran at 40.0 kV and 30 mA. According to specified criteria, the exposure period was chosen, and phase identification was carried out using search-match software using the Joint Committee on Powder Diffraction- International Centre for Diffraction Data ( JCPDS-ICDD) files as a reference.

### Analysis of Alkalinizing Activity (pH)

Polypropylene tubes with an internal diameter of 2 mm and a height of 10 mm were used for the investigation of alkalinizing activity (pH), with both ends left open. Prior to filling the tubes with the material, their weights were measured. The material was then thoroughly mixed in accordance with the manufacturer's instructions. After the powder of the examined substance was mixed in various amounts with SeNPs powder to gauge its alkalinizing action. Each of the five groups in the experiment had a different concentration of nanoparticles:

Group 1 (control): 0% w/w SeNPs

Group 2: 0.5% w/w SeNPs

Group 3: 1% w/w SeNPs

Group 4: 1.5% w/w SeNPs

Group 5: 2% w/w SeNPs

The freshly mixed material was placed within tubes. The exterior walls were gently cleansed from any extra debris using moistened gauze. To guarantee a consistent amount of substance in each tube, the filled tubes were then weighed on a very precise balance (0.0001 g) ^12^. For each experimental group, a total of five specimens were created. A negative control group of empty tubes (n = 5) was used in the study to validate the approach. After the complete setting of the material, each filled tube (specimen) was promptly immersed in an individual plastic container containing 10 ml of distilled water with a pH of 6.5. The containers were closed and placed in an incubator set at 37°C and relative humidity throughout the experimental duration ^13^.

Each tube was removed from its original container at the end of each experimental incubation period and immediately placed in a new container with 10 ml of freshly distilled water. To determine whether the pH values of the pulp capping materials were affected by time, this process was repeated at 1, 7, 14, and 30 days ^14^. The pH of the solution within each containerwas measured at each time interval using a digital pH meter that had previously been calibrated with standard solutions at pH 4, 7, and 10, and the measurements were conducted at a temperature of (25°C) ^12^^,^^13^. After removing the material, the container underwent shaking for 5 seconds to achieve uniform hydroxyl ion distribution. To achieve reliable measurements, the pH-meter electrode was soaked in each container and allowed to stabilize. To avoid unreliable results, stirring was done to achieve uniform contact with the electrode tip. Care was also taken to avoid any contact with the container's base or the creation of air bubbles. The pH-meter electrode was cleaned with distilled water after each usage and dried with a paper towel to avoid contamination from the prior solution. The pH readings were taken continuously at a temperature of 25°C ^12^^,^^13^. For comparison over time, the mean pH values of each group and the control group were calculated and recorded. As a result, we were able to calculate the alkalinizing activity of the soaking water for each incubation period and assess how it changed over time.

### Statistical Evaluation

The SPSS software version 22 for Windows was used to analyze the study's data. The Shapiro-Wilk test was used to assess normality, with a significance level of P≤ 0.05 used for the test data. Given that the pH data have a normal distribution, parametric statistical tests have been used. All of the data that was gathered underwent descriptive analysis, which included mean and standard deviation. One-way analysis of variance (ANOVA) was used in the pH study to find significant differences between the groups. The purpose of Tukey's test was to find whether the group has statistically significant differences from the others among multiple comparisons. The results for pH were interpreted using a constant significance level of P≤0.05 throughout the analyses.

## Results

### Characterization of Hydrated MTA Repair HP

**Figure 1:**
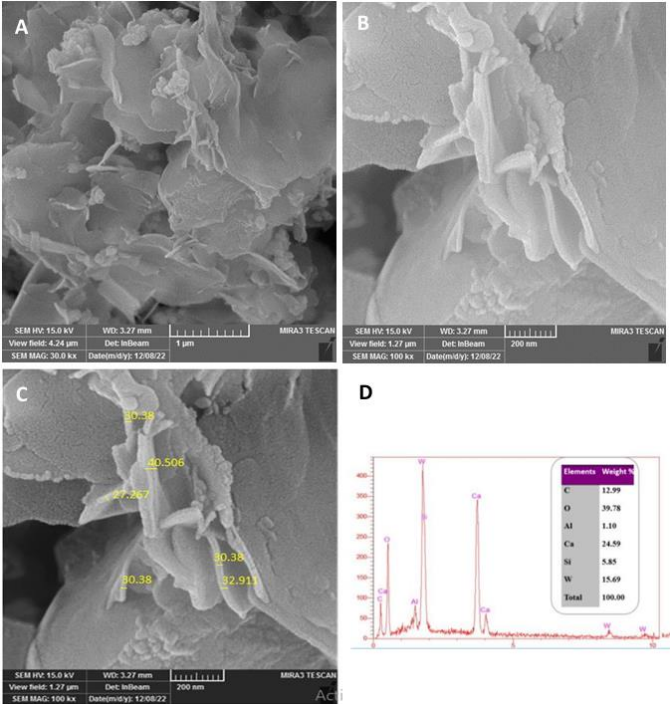
shows an EDX spectrum and a FESEM image of hydrated MTA Repair HP after setting but before integrating selenium nanoparticles

Using FEG-SEM observations and EDX analyses, the microstructural characteristics of MTA Repair HP were examined. The FEG-SEM observations allowed for the examination of morphological changes that occurred following the setting procedure and provided visual insights into the material's structure. The EDX analysis, on the other hand, allowed for the determination of the chemical constituents of the substance. The FEG-SEM study of MTA Repair revealed the presence of a plate-like shape with a variety of features (Figure 1). These features were found to be randomly distributed across the surface of the material, and they could be differentiated by their nano-scale diameters. This random distribution emphasizes how the microstructure of the material is heterogeneous and the features at the nanoscale are not structured in any particular or systematic pattern. Such a complex and variable distribution of nanometric characteristics contributes to the hydrated MTA HP material's distinctive qualities and behavior. EDX analysis was used to determine the chemical composition of the MTA HP material. According to the findings, the substance was predominantly composed of the following components, in varied weight percentages: silicon (5.85%), aluminum (1.10%), calcium (24.59%), tungsten (15.69%), and oxygen (39.78%). These findings help to a thorough understanding of the material's elemental composition by highlighting the existence and relative abundance of distinct elements. These results lead to a deeper comprehension of the characteristics and behavior of MTA HP, which is useful for its application in a variety of industries, including dentistry.

### SeNPs Characterization

**Figure 2:**
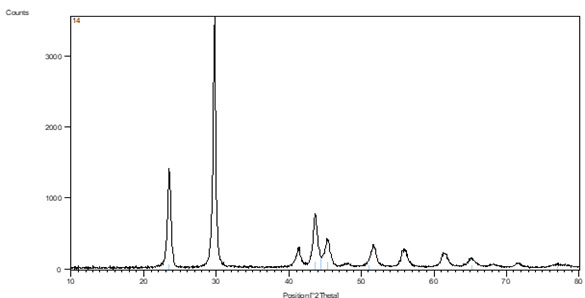
SeNPs XRD pattern

As part of the characterization approach, the crystal structure, lattice constants, and average crystalline size of SeNPs were investigated using X-ray diffraction (XRD) spectrum analysis. According to the XRD analysis, the SeNPs' (100), (101), (110), (102), (111), (200), (201), (003), (112), (103), (210), (211), and (113) planes all had distinct diffraction peaks (Figure 2). These peaks matched the typical ICDD card number (00-006-0362) and proved the SeNPs' hexagonal structure (Table 1).

**Table 1:** Shows the 2-theta for the main distinctive peaks of SeNPs powder

2-Theta (θ)	FWHM	D (Crystal size in nm)
23.51	0.1428	56.82401
29.70	0.1636	50.23714
41.30	0.1639	51.79827
43.64	0.1528	56.00374
45.35	0.1720	50.0568
51.72	0.1724	51.20845
56.14	0.1852	48.61403
Mean		52.10606

The Full Width at Half Maximum (FWHM) indicated the degree of peak broadening, whilst the angles of diffraction (given as 2-Theta values) provided information about the crystal arrangement. This broadening was a sign of defects in the crystalline structure and SeNP size. The crystal dimension (D), which ranged from 48.6 nm to 56.8 nm with an average measurement of about 52 nm, could be identified using the Debye-Scherrer equation on the FWHM data. These findings offer important information about the size of the SeNPs created using environmentally friendly methods, which is essential for understanding their characteristics and prospective applications. The effectiveness and potential of the nanoparticles are greatly influenced by the careful control of crystal size and structure.

**Figure 3:**
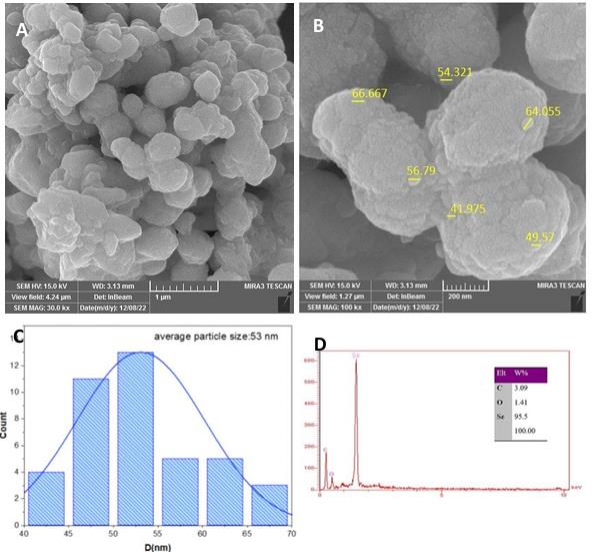
Displays the particle size distributions and related EDX spectrum for a selenium nanoparticle captured using a FESEM

The morphology of SeNPs was studied using FESEM. The observed formations were mostly spherical, with some aggregation zones. As shown in Figure 3, the subsequent particle size assessment generated from FESEM image analysis validated dimensions ranging from 41.9 to 66.6 nm, with an average particle size of 53 nm. These outcomes support the conclusions drawn from XRD analysis, showing a coherent grain size trend that is in line with the XRD data. EDX analysis was used to determine the elemental composition of the nanoparticles. Figure 3 shows the EDX spectrum, which clearly shows a distinct and strong peak at 1.5 keV that corresponds to SeNPs. Additionally, signals attributable to Carbon (C) and Oxygen (O), which are relatively weaker signals, were also detected. These findings support the excellent purity of the SeNPs. (Figure 3) These comprehensive results highlight the purity of the SeNPs and enable us to understand their morphological and elemental features better.

### Hydrated MTA HP Repair Analyses Following Selenium Nanoparticle Incorporation

**Figure 4:**
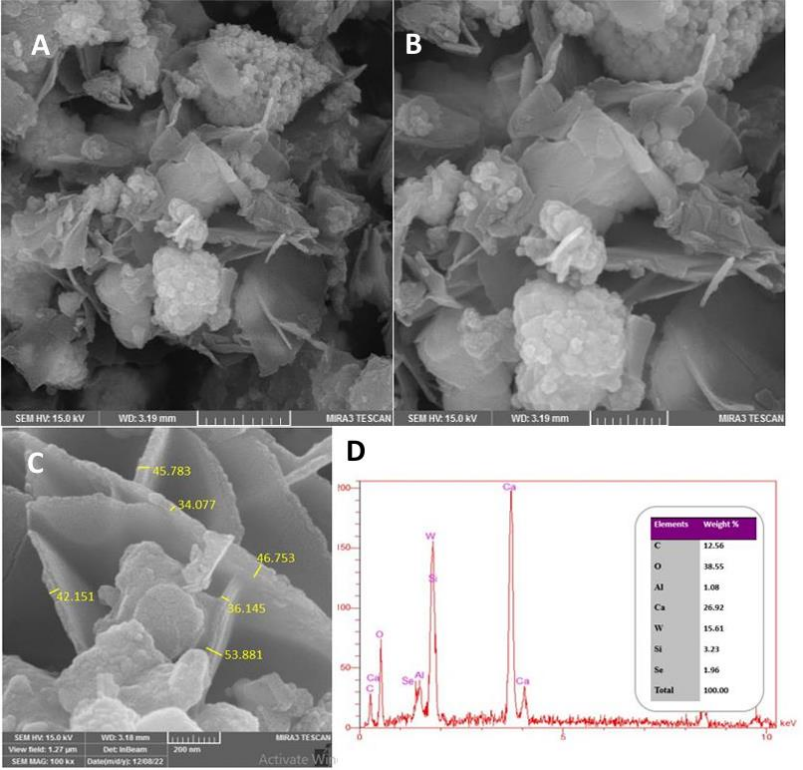
EDX spectra and a FESEM image of a hydrated MTA Repair HP with SeNPs.

It was clear from FE-SEM analysis that SeNPs had been successfully incorporated into the MTA HP matrix by way of specific morphological characteristics. The micrographs showed a dispersion of nanoparticles grouped or arranged into clusters or agglomerates. This clustering phenomenon shows that SeNPs were successfully dispersed and integrated into the MTA HP matrix. (Figure 4.)

Selenium (Se) was identified by EDX analysis as a distinctive element, verifying the efficient integration procedure. Furthermore, the weight % of various chemical components in the hydrated MTA HP integrated with SeNPs was determined. Aluminium (Al) 1.08%, Calcium (Ca), Tungsten (W) 15.61%, Silicon (Si) 3.23%, and Selenium (Se) 1.96% were all present in the composition, along with Carbon (C) 12.56%, Oxygen (O) 38.55%, Silicon (Si) 3.23%, and Aluminium (Al). The EDX study also showed alterations in the elemental ratios. The weight percentage of Silicon (Si) reduced from 5.85% to 3.23% after SeNPs were included in the hydrated MTA HP, whereas the weight percentage of Calcium (Ca) increased from 24.59% to 27.01%. These alterations provide information about the chemical changes occurring within the integrated substance.

### pH Measurement Analysis

**Figure 5:**
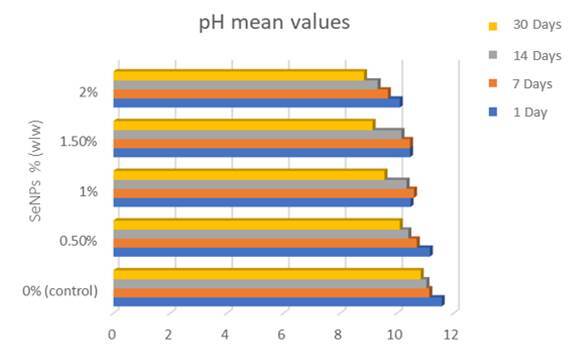
A bar graph showing the average pH values over time for the MTA repair HP group and the experimental groups with various SeNPs concentrations

**Figure 6:**
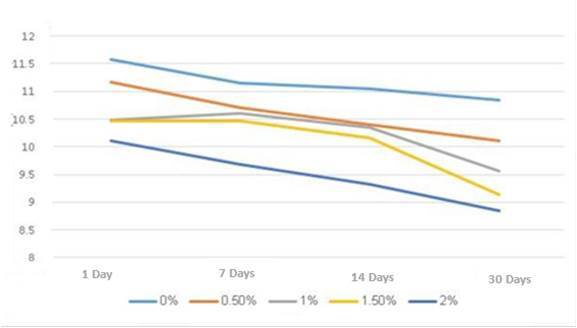
The average pH values over time for the MTA repair HP group and experimental groups subjected to various SeNPs concentrations

The collected data underwent statistical analysis and showed significant differences in the alkalinizing activity using the one-way analysis of variance (ANOVA) method for the pH value of the tested groups after each storage period at (P≤0.05) Table 2. In addition, for each group after different storage periods at (P≤0.05) Table 3. Followed by Tukey's post hoc test for conducting multiple comparisons between the groups Table 4 and Figure (5 and 6), which showed the means and standard deviation of pH values for the MTA Repair HP that were calculated for two scenarios: before and after incorporating different concentrations of (SeNPs) at the different storage periods that varied significantly at (P≤0.05). The investigation revealed a discernible reduction in the alkalinizing activity of the control group with each incorporated concentration of (SeNPs) that significantly differed from the control group across various periods. Consequently, the findings indicate that the addition of SeNPs to MTA HP has a notable impact on the pH of the storage solution, leading to a significant decrease in pH values for all concentrations and periods when compared to the control group.

**Table 2:** Analysis of variance (ANOVA) for the pH value of the tested material groups after each storage period.

Periods		Sum of Squares	df^*^	Mean Square	F- value	P-value**
1 Day	Between Groups	7.182	4	1.795	71.816	0.000
	Within Groups	.500	20	0.025		
	Total	7.682	24			
7 Day	Between Groups	5.662	4	1.415	96.945	0.000
	Within Groups	.292	20	0.015		
	Total	5.954	24			
14 Day	Between Groups	7.638	4	1.910	21.408	.000
	Within Groups	1.784	20	0.089		
	Total	9.422	24			
30 Day	Between Groups	12.662	4	3.165	58.188	.000
	Within Groups	1.088	20	0.054		
	Total	13.750	24			

**sig. value=0.000

**Table 3:** Analysis of variance (ANOVA) for the pH value of each group after different storage periods.

Periods		Sum of Squares	df^*^	Mean Square	F-value	P-value**
0% (control)	Between Groups	1.466	3	.489	10.799	0.000
	Within Groups	.724	16	.045		
	Total	2.190	19			
0.5%	Between Groups	3.066	3	1.022	14.445	0.000
	Within Groups	1.132	16	.071		
	Total	4.198	19			
1%	Between Groups	3.298	3	1.099	17.728	.000
	Within Groups	.992	16	.062		
	Total	4.290	19			
1.5%	Between Groups	5.882	3	1.961	64.279	.000
	Within Groups	.488	16	.030		
	Total	6.370	19			
2%	Between Groups	4.297	3	1.432		.000
	Within Groups	.328	16	.021		
	Total	4.625	19		69.878	

**sig. value=0.000

**Table 4:** shows the mean and standard deviation of pH values taken at various time intervals

SeNPs (%)	Storage periods (Mean and SD)			
	1 Day	7 Days	14 Days	30 Days
0% (control)	11.58 ± 0.130^b, D^	11.14 ± 0.134^a, D^	11.04 ± 0.364^a, C^	10.84 ± 0.114^a, D^
0.5%	11.16 ± 0.167^c, C^	10.70 ± 0.100^c, b, C^	10.40 ± 0.441^b, a, B^	10.10 ± 0.223^a, C^
1%	10.48 ± 0.148^b,B^	10.60 ± 0.122^b,B,C^	10.34 ± 0.207^b,B^	9.56 ± 0.409^a,B^
1.5%	10.46 ± 0.134^b,B^	10.46 ± 0.114^b,B^	10.16 ± 0.270^b,B^	9.14 ± 0.134^a, A, B^
2%	10.10 ± 0.200^d, A^	9.68 ± 0.130^c, A^	9.32 ± 0.044^b, A^	8.84 ± 0.151^a, A^

In the present study, the variable SD (representing the standard deviation) was utilized. The use of distinct superscript capital letters (A-D) to label values indicated a statistically significant distinction between groups at varying concentrations of SeNPs (%) but within the same storage period (within the same column). Furthermore, the use of different superscript lowercase letters (a-d) to annotate values demonstrated a statistically significant difference between groups with the same concentration of SeNPs but at different storage periods (within the same row), when compared to the control group (with a significance level of P≤0.05).

The findings elucidated that the control group yielded a high pH level (11.58) on the first day of storage solution. After this, a statistically significant reduction in its alkalinizing efficacy, 11.14, 11.04, and 10.84 at the subsequent periods respectively was observed compared to the pH levels recorded on the first day, with no statistically discernible differences noted across subsequent time intervals (p > 0.05). Specifically, at a SeNPs concentration of 0.5%, no statistically significant disparities were noted in pH values between the 1-day and 7-day intervals. Furthermore, analogous non-significant differences were observed between the 7-day and 14-day periods. Moreover, the comparison of pH values between the 14-day and 30-day intervals exhibited no statistically significant variances. There was no statistically significant difference in pH values among days 1, 7, and 14, which were distinct from the values observed on day 28 for each 1% and 1.5% groups. Conversely, the 2% group exhibited significant variations in pH values across all assessed time intervals.

## Discussion

The FESEM/EDX results for hydrated MTA HP analyses offer important details on the material's microstructure and elemental composition. This study's FESEM observations showed the formation of plate-like crystals in varied thicknesses and random directions, which is compatible with the results described in the literature ^15^. This supports the reaction of calcium silicates and water and indicates that the crystal growth pattern is a distinctive feature of hydrated MTA HP(). Aluminium (Al), Silicon (Si), and calcium (Ca) were identified in the elemental analyses performed using EDX technology. This shows that the particles contain all three phases, Ca_3_SiO_5_, Ca_2_SiO_4_, and Ca_3_Al_2_O_6_, which were previously found using X-ray diffraction examination ^16^. The identification of these elements validates the composition of MTA HP and adds to our understanding of its chemical structure.

A previous study by Jiménez-Sánchez et al. (2019) has also demonstrated the significant calcium (Ca) content of MTA HP and small amounts of silicon ^17^. These results support the elemental composition identified in the current investigation and provide additional evidence that these elements are present in hydrated MTA HP.

The detection of Carbon following the setting of MTA HP as a consequence of the carbonization of CaO during sample preparation for FESEM analysis, as well as the production of calcium carbonate as a result of the reaction between calcium hydroxyl and ambient carbon dioxide (CO_2_) in the sample. This result is in line with studies by Camilleri (2014), which established that calcium carbonate is produced during the carbonation of hydrated cement by the reaction of calcium oxide with surrounding carbon dioxide ^17^.

The investigation showed that the nanoparticles had an almost spherical shape. With an average particle size of about 53 nm, the particle size distribution showed a wide range. The high-resolution images acquired from FESEM enabled a detailed investigation of the surface morphology, which revealed a rough texture and aggregation regions. In addition, the elemental composition of the SeNPs was confirmed by EDX analysis. The EDX spectrum exhibited an intense peak at 1.5 keV corresponding to selenium ^18^. The finding of selenium in the spectrum is further evidence for the SeNPs' purity and crystalline properties.

The lack of notable peaks for other elements shows that the SeNPs were extremely pure. Carbon (C) signals found in the FESEM analysis in the current investigation can be attributed to the presence of this element on the sample grid used for imaging. Similar to this, the oxygen (O) signals found in the EDX spectrum during sample preparation and analysis most likely came from the surrounding atmosphere ^19^. The unique peaks in the spectrum corresponding to oxygen provide further confirmation that the nanoparticles under research do not exist in their oxide state, but rather as pure selenium nanoparticles ^20^. This observation supports the study's successful pure selenium nanoparticle characterization by confirming the selenium-dominant elemental composition.

The insertion of SeNPs into MTA HP can result in a variety of morphological modifications in the material. This article seeks to describe prospective results from FESEM and EDX analysis, offering insight into potential microstructural and elemental changes. The FESEM analysis is used to detect morphological changes caused by the integration of SeNPs. It displays the dispersion of SeNPs within the MTA HP matrix, where the nanoparticles tend to aggregate or cluster. This observation shows that SeNPs were successfully incorporated into the substance. Such inclusion may result in distinctive morphological characteristics, which may affect the material's surface characteristics and interactions. In addition, surface alterations brought on by SeNPs are seen in FESEM measurements. When compared to the non-nanoparticle-incorporated MTA HP, the nanoparticles introduce surface characteristics such as roughness or protrusions. The interactions of the material with its surroundings, such as adhesion to other materials or the biological environment, can be profoundly impacted by these changes in surface roughness. Additionally, SeNPs inclusion alters the pore structure and porosity of MTA HP. The nanoparticles affect the material's overall porosity by filling in or partially blocking the existing pores. Furthermore, their presence can impact the development of new pores or change the connectivity and size distribution of existing ones.

EDX analysis enables the identification of components present in the MTA HP in the context of integrating SeNPs. The presence of selenium (Se) demonstrates that the nanoparticles were successfully incorporated. Additionally, the altered elemental ratios-in particular, increasing calcium and decreasing silicon contents-indicate possible chemical interactions between selenium and the MTA matrix. The greater mechanical strength and improved biocompatibility of the inserted material may be attributed to these interactions. The results of this study offer important new understandings of the morphological and elemental alterations in hydrated MTA HP that take place when SeNPs are added. These findings advance knowledge of the qualities and prospective performance of the integrated material, which affects how well it may be used in dental operations.

MTA has several uses in clinical settings, including pulp capping, root perforation healing, and root end filling. The pH levels may differ when utilized close to tissue fluids like serum and blood because of infection and inflammation. MTA releases calcium and hydroxyl ions from its calcium hydroxide (Ca(OH)2) molecule upon coming into touch with tissue fluids, causing the pH to rise to about 12.5 ^21^. 

The presence of calcium ions and these alkaline pH levels in the tissues surrounding the MTA is essential for fostering the formation of hard tissues. The surrounding tissues, however, may be harmed by the prolonged and strong alkalinity of MTA ^22^. The current study sought to solve this by introducing nanomaterials renowned for their antibacterial, anti-inflammatory, and antioxidant capabilities to lower the pH of hydrated MTAHP.

 SeNPs were chosen for their superior biological and physical properties. They have antibacterial action that prevents microbial growth as well as antioxidative qualities that fight the impacts of free radicals and reactive oxygen species (ROS). Additionally, SeNPs have shown strong immunomodulatory properties that affect immune cells and related signaling processes ^23^.

There are numerous methods available for determining pH in vitro. The pH of the samples was measured using distal water in Polypropylene tubes. The findings showed that MTA Repair HP consistently had high alkaline pH values at various time intervals. The alkaline pH is related to the dissociation of calcium oxide, which is found in MTA, into calcium hydroxide and OH- ions ^2^^,^^24^. The rate of calcium hydroxide generation can be connected to changes in pH over time ^24^.

MTA produces calcium hydroxide when it comes into touch with water or bodily fluids, which raises the pH of the environment by releasing hydroxyl ions ^6^.

Therefore, damage provoked adjacent tissues, to high and long-term alkalinity ^8^. The initial alkaline pH reported in MTA-HP is consistent with prior observations ^25^. Alkalinity decreased over time after SeNPs were added to MTAHP in a concentration-dependent manner and pH variations were observed after 1, 7, 14, and 30 days. It can be difficult to compare the findings of this study's hydrated MTA HP pH before SeNPs incorporation with those of other studies because of methodological variations. To guarantee standardized test conditions and data comparability, the study used distilled water with a pH of less than 7 (6.5), which is comparable with earlier investigations ^13^. After measuring pH, the samples were put in a fresh solution to avoid medium saturation. This strategy is consistent with the usage of routine medium exchange to preserve stable conditions ^14^. The MTA HP demonstrated sustained high alkalinizing activity throughout the testing periods, despite an overall decline in alkalinizing activity over time. Similar trends have been observed in previous experiments, demonstrating MTA's ability to maintain a raised pH over extended periods ^2^^,^^26^. The findings of this study support the initial hypothesis, confirming that SeNPs indeed influence the alkalinizing activity of MTA HP. To comprehensively assess the impact of SeNPs, further investigations are warranted to explore their effects on various physicochemical properties of MTA pulp capping material.

## Conclusions

In conclusion, our investigation has shed light on the profound impact of nano selenium on the pH of mineral trioxide aggregate (MTA). The noticeable decrease in pH, as compared to MTA without nano selenium, carries significant implications for the preservation of pulp tissue. Considering MTA's inherent high pH and its potential association with superficial necrosis, the incorporation of nano selenium emerges as a promising approach to counteract this undesirable effect. As a result, our research underscores the substantial benefits of utilizing nano selenium, and we anticipate that these findings will contribute to the advancement of biomedical materials and therapies.
